# Effects of emergency obstetric care training on maternal and perinatal outcomes: a stepped wedge cluster randomised trial in South Africa

**DOI:** 10.1136/bmjgh-2019-001670

**Published:** 2019-11-10

**Authors:** Nynke van den Broek, Charles Ameh, Barbara Madaj, Jennifer Makin, Sarah White, Karla Hemming, J Moodley, Robert Pattinson

**Affiliations:** 1 Centre for Maternal and Newborn Health, Liverpool School of Tropical Medicine, Liverpool, UK; 2 Department of Obstetrics & Gynaecology, University of Pretoria, Pretoria, South Africa; 3 Institute of Applied Health Research, University of Birmingham, Birmingham, UK; 4 Womens Health and HIV Reaserch unit, University of KwaZulu Natal, Durban, South Africa; 5 MRC Maternal and Infant Health Care Strategies Unit, University of Pretoria, Pretoria, South Africa

**Keywords:** maternal health, obstetrics, health services research, health systems evaluation

## Abstract

**Introduction:**

Two-thirds of maternal deaths and 40% of intrapartum-related neonatal deaths are thought to be preventable through emergency obstetric and newborn care (EmOC&NC). The effectiveness of ‘skills and drills’ training of maternity staff in EmOC&NC was evaluated.

**Methods:**

Implementation research using a stepped wedge cluster randomised trial including 127 of 129 healthcare facilities (HCFs) across the 11 districts in South Africa with the highest maternal mortality. The sequence in which all districts received EmOC&NC training was randomised but could not be blinded. The timing of training resulted in 10 districts providing data before and 10 providing data after EmOC&NC training. Primary outcome measures derived for HCFs are as follows: stillbirth rate (SBR), early neonatal death (ENND) rate, institutional maternal mortality ratio (iMMR) and direct obstetric case fatality rate (CFR), number of complications recognised and managed and CFR by complication.

**Results:**

At baseline, median SBR (per 1000 births) and ENND rate (per 1000 live births) were 9 (IQR 0–28) and 0 (IQR 0–9). No significant changes following training in EmOC&NC were detected for any of the stated outcomes: SBR (adjusted incidence rate ratio (aIRR) 0.97, 95% CI 0.91 to 1.05), iMMR (aIRR 1.23, 95% CI 0.80 to 1.90), ENND rate (aIRR 1.04, 95% CI 0.92 to 1.17) and direct obstetric CFR (aIRR 1.15, 95% CI 0.66 to 2.02). The number of women who were recognised to need and received EmOC was significantly increased overall (aIRR 1.14, 95% CI 1.02 to 1.27), for haemorrhage (aIRR 1.31, 95% CI 1.13 to 1.52) and for postpartum sepsis (aIRR 1.86, 95% CI 1.17 to 2.95)

**Conclusion:**

Following EmOC&NC training, healthcare providers are more able to recognise and manage complications at time of birth. This trial did not provide evidence that the intervention was effective in reducing adverse clinical outcomes, but demonstrates randomised evaluations are feasible in implementation research.

**Trial registration number:**

ISRCTN11224105.

**Key questions**

**What is already known?**

- ‘Skills and drills’ training in emergency obstetric and newborn care (EmOC&NC) improves knowledge and skills of health providers.
- Training of healthcare providers is a substantial component of the majority of programmes in low-income and middle-income countries which seek to improve maternal and perinatal health outcomes.

**What are the new findings?**

- A new type of trial design—the stepped wedge trial—was used to conduct implementation research to assess the effectiveness of training in emergency obstetric and newborn care (EmOC&NC) on health outcomes.
- Maternal case fatality rates and stillbirth rates did not significantly reduce but a significant increase was noted in the number of women recognised by healthcare providers to need, and, who received, emergency obstetric care.

**What do the new findings imply?**

- A stepped-wedge trial design allows for rigorous evaluation of complex interventions in real life settings.
- Proxy measures are needed to serve as outcomes in settings where the absolute number of maternal deaths has declined.

## Introduction

An estimated 300 000 women worldwide die from complications of pregnancy and childbirth.^1^ In addition, an estimated 2.6 million stillbirths and 2.8 million neonatal deaths occur each year, the latter accounting for at least 45% of deaths in children aged less than 5 years.^2 3^ The majority of these deaths could be prevented or avoided through actions that are proven to be effective and affordable. More than 50% of stillbirths occur at the time of birth and/or are associated with maternal emergencies.^4^ Neonatal deaths are the result of complications of prematurity (35%), intrapartum-related complications (including asphyxia; 24%), sepsis (15%), congenital abnormalities (10%) and pneumonia (5%).^3^ The majority of maternal deaths (up to 80%) which occur globally are direct maternal deaths. For these, there are five main groups of causes—obstetric haemorrhage (27% of all maternal deaths), hypertensive disorders (eclampsia and pre-eclampsia) (14%), sepsis or infection (11%), and complications of obstructed labour (9%) and abortion (8%).^3^

There are evidence-based highly effective interventions agreed for each of these complications during or after pregnancy.^5^ In 1997, the key interventions needed were bundled into a care package known as emergency obstetric care (EmOC) (box 1).^6^

Box 1 **Levels of emergency obstetric care and their signal functions**

Basic emergency obstetric care

- Provide intravenous/intramuscular antibiotics.
- Provide intravenous/intramuscular oxytocics.
- Provide intravenous/intramuscular anticonvulsants.
- Manual removal of a retained placenta.
- Removal of retained products of conception (eg, using manual vacuum aspiration).
- Assisted vaginal delivery (eg, vacuum extraction).
- Perform basic neonatal resuscitation (eg, with bag and mask).

All seven Basic EmOC functions above, plus:

- Caesarean section.
- Blood transfusion.

The estimated maternal mortality ratio (MMR) in 2015 for the Republic of South Africa was 138/100 000 live births, although considerable disparities in MMR by province exist.^3 7^ In South Africa, indirect deaths are more common than direct obstetric deaths, and non-pregnancy infections (mostly HIV related), haemorrhage and hypertension together account for 70% of maternal deaths.

South Africa is one of the few countries in sub-Saharan Africa with a well-established Confidential Enquiry into Maternal Deaths (CEMDs), which was started in 1998 and has published the seventh triennial report (2014–2016) in 2018. For South Africa, it was estimated that up to 60% of maternal deaths and 40% of asphyxia-related neonatal deaths could be prevented if healthcare providers had the necessary knowledge and skills supported by a fully functional health system to provide quality EmOC.^8 9^ One of the recommendations following the CEMD report in 2008 was that all maternity care providers should receive training in EmOC and that ‘fire drills’ should be introduced in maternity wards.^10^

Short ‘skills and drills’ type simulation training has been extensively evaluated and it is known to improve healthcare providers’ knowledge and skills, as well as the availability of EmOC signal functions. However, there is far less evidence that training results in a reduction in maternal and/or perinatal mortality and morbidity.^11–14^

A cross-sectional stepped wedge design (SWD), cluster randomised trial was conducted in the 11 districts of South Africa with the highest maternal mortality. The objective of the study was to assess the effectiveness of ‘skills and drills’ training workshops in EmOC&NC for healthcare providers providing maternity care in healthcare facilities (HCFs), by considering four outcomes at HCF level: stillbirth rate (SBR), early neonatal death (ENND) rate, institutional MMR (iMMR) and direct obstetric case fatality rate (CFR: number of maternal deaths over number of women with complications).

## Methods

A stepped-wedge cluster randomised trial to assess the effectiveness of ‘skills and drills’ training in EmOC was conducted in 11 districts in South Africa with the highest levels of maternal mortality. This evaluation was based on a planned evaluation, commissioned by stakeholders, and the sample size was thus dictated by the number of clusters available for inclusion and the number of observations within each cluster dictated by the number of births over the 26-month study period. In line with recommendations, no post hoc power calculation was performed.^15^

### Study sites and implementation schedule

**Figure 1:**
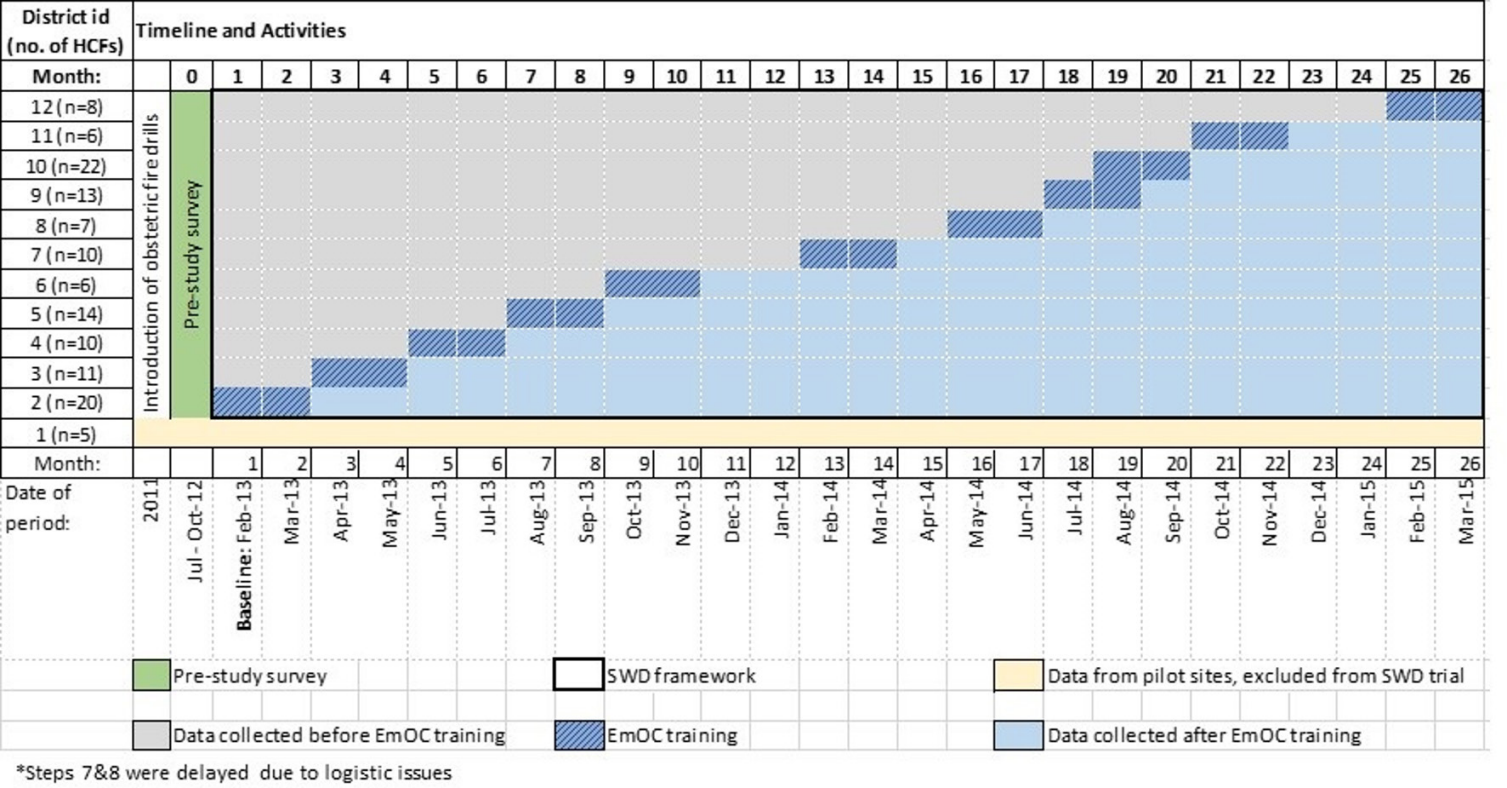
Schematic representation of the stepped wedge, cluster randomised trial implementation. EmOC, Emergency Obstetric Care; HCFs, healthcare facilities; SWD, stepped-wedge, cluster randomised trial.

About 50% of all maternal deaths in South Africa occur in 12 of the 45 districts that do not have a medical school; these districts were selected for the trial. (Medical schools were already providing EmOC training, thus the seven districts with medical schools were excluded.) One district (with five HCFs) was randomly selected to be a designated pilot site and therefore also excluded from the study resulting in a total of 11 districts in which the trial was conducted. HCFs are categorised according to their designation to provide either basic or comprehensive EmOC (box 1). HCFs were clustered by district for randomisation, delivery of the intervention and the analysis. The sequence of implementing the intervention was determined using simple random sampling by an independent person drawing folded pieces of paper bearing the 11 district names from a hat, which was documented. The order in which the districts were sampled constituted the order of the randomised roll-out. EmOC&NC training workshops were delivered one district at a time over a period of two calendar months per district between February 2013 and March 2015. These 2-month roll-out periods were defined as transition periods and data for these were not included within the study evaluation. The months of December and January were not used as training periods as these are holiday months in South Africa (figure 1).

### Intervention

At the time of commencing the trial for all HCFs providing basic or comprehensive EmOC, standardised obstetric ‘fire drills’ were being conducted on each labour ward each month by trained clinical leads which consisted of a lecture, skills training, fire drill, assessment of drill and feedback.

In addition to this, ‘skills and drills’ training workshops in EmOC&NC were introduced and delivered over 2 days (junior midwives) or 3 days (senior midwives and all medical staff) off-site, for at least 80% of all staff involved in providing maternity services for each participating HCF.

The training package used in RSA was adapted from the Life Saving Skills – Essential (Emergency) Obstetric and Newborn Care (EmOC&NC) training programme developed by the Centre for Maternal and Newborn Health at the Liverpool School for Tropical Medicine.^16 17^ The EmOC&NC training workshop content covers the essential knowledge and skills required by skilled birth attendants to recognise and manage the major causes of maternal and newborn death in low-income and middle-income (LMIC) and includes all EmOC signal functions^6^; maternal and newborn resuscitation, early newborn care (recognition and management of prematurity, hypoglycaemia and hypothermia), communication triage and referral, management of shock and the unconscious patient, recognition and management of severe pre-eclampsia and eclampsia, recognition, prevention and management of obstetric haemorrhage, sepsis, use of the partograph, recognition and management of obstructed labour, ability to perform assisted vaginal delivery (ventouse delivery), manual removal of retained placenta and manual vacuum aspiration for retained products of conception, recognition and management of other obstetric emergencies (breech delivery, cord prolapse, twin delivery, shoulder dystocia) and managing difficult caesarean sections.^16^ For South Africa, additional modules included the recognition and management of complications in women who are HIV positive.

### Outcome measures

The protocol was registered retrospectively at the time when it became possible to register step-wedge designed trials; maternal and newborn mortality were identified as societal outcomes to be assessed. Each outcome defined involved a ratio of two counts. The primary outcome measures, at HCF level, identified in the ISRCTN registration were SBR (stillbirths per 1000 births), ENND rate (ENND per 1000 live births), institutional maternal mortality ratio (iMMR per 100 000 live births) and direct obstetric case fatality rate (CFR—number of maternal deaths due to direct obstetric complications as a proportion of the number of women with direct obstetric complications). The secondary outcome measures assessed though not specified in the registered protocol include obstetric CFRs for all complications and for indirect causes only, direct and indirect institutional maternal deaths, and CFR specifically for obstetric haemorrhage, postpartum haemorrhage (PPH), (pre-)eclampsia, postpartum sepsis, obstructed labour and ruptured uterus. Subsequent to analyses of these outcomes, complication rates (number of women attending for birth recognised to require management for each complication as a proportion of the number of live births) were also analysed (see online supplementary table 1).

10.1136/bmjgh-2019-001670.supp1 Supplementary data

### Data collection

Data required to derive the selected outcomes were collected monthly (by phone or in person by a member of the research team) over the study period from HCF registers using a standardised data extraction tool. In addition, data were processed and checked during field visits with at least two visits for each included HCF during the study. Outstanding anomalies in the data were addressed (see online supplementary table 2).

10.1136/bmjgh-2019-001670.supp2 Supplementary data

### Statistical methods

#### Baseline descriptive summary

Data for month 1 (February 2013) were used as baseline data for this study. Summary statistics for the outcomes analysed were derived for each facility type and overall. To classify the underlying workload of each facility, baseline data for the number of births recorded were used; initially three quartile values were used as boundary values to define four workload categories. Since use of this categorical variable in initial analysis of SBRs detected no difference between the middle two categories, these were merged, resulting in categorisation of facilities into: low volume, with ≤30 births/month; medium volume, with 31–160 births/month; or high volume, HCFs with >160 births/month.

#### Analyses of outcomes

Allowed for the clustering, confounding effects of time inherent with the SWD, and overdispersion of count outcomes. To this end, mixed effects (also known as multilevel) negative binomial regression models (log link), with robust standard errors, were used to examine the evidence of intervention effects. For SBR, the response used for each facility each month was the number of stillbirths, and the number of births was the offset. For ENNDs and institutional maternal mortality, the offset was the number of live births, and for CFRs, the offset was the number of reported complications specific to the cause of death. For complication rates, the offset was the number of births. The effect of the intervention is therefore reported as an incidence rate ratio (IRR), with 95% CI. The analyses adjusted for month of study to account for both any underlying seasonal variation and any secular trends, facility type (basic or comprehensive), baseline facility workload (defined using interquartile values: low volume (≤30 births/month); medium volume (31–160 births/month) or high volume (>160 births/month)), prestudy district MMR (2011–2012) and additionally included random effects for district and facility. To examine evidence of a differential impact of the intervention associated with risk factors, interactions between intervention and each of facility workload category, facility type and district were considered in sensitivity analyses. For each cluster, the 2 months in which the intervention was introduced was regarded as transition months and was therefore excluded from analyses (a decision made prior to data analysis commencing). Data for a facility were omitted from analysis when values were missing for either the outcome or a covariate required for the analysis. Analyses were performed as implemented.

### Patient and public involvement

No patients were involved in this study.

## Results

**Figure 2:**
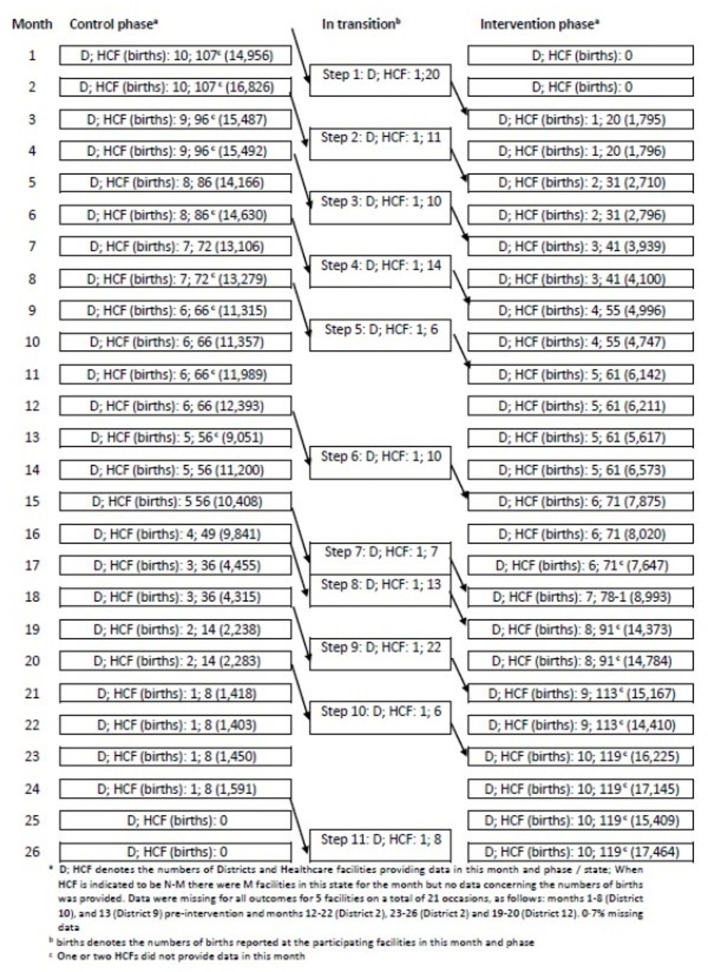
Consolidated Standards of Reporting Trials flow diagram for stepped wedge, cluster randomised trial. HCF, healthcare facility.

Two HCFs did not submit any data and were therefore excluded from analysis, resulting in a total of 127 HCFs from 11 districts, of which 75 were designated to provide comprehensive EmOC (figure 2). The number of facilities per district varied from 6 to 22.

### Characteristics of participating facilities

The characteristics of the participating facilities at month one are described in table 1. The median number of births occurring per month was 35 (IQR 20–70) in HCFs designated to provide basic EmOC and 103 (IQR 52–246) in HCFs designated to provide comprehensive EmOC. The total number of births during this month was 16 508; 83% (13 736) of which were at comprehensive EmOC facilities. All districts had at least one comprehensive EmOC facility within a high workload category.

**Table 1:** Characteristics of included healthcare facilities by healthcare facility type (designated to provide basic or comprehensive emergency obstetric care)

Characteristics	Basic (n=52)	Comprehensive (n=75)	All (n=127)
Workload category: n (%)			
1–30 births per month	22 (42%)	9 (12%)	31 (24%)
31–160 births per month	25 (48%)	36 (48%)	61 (48%)
>160 births per month	4 (8%)	30 (40%)	34 (27%)
No. of births per month: Median (IQR)	35 (20–70)	103 (52–246)	64 (31–178)
No. of live births per month: Median (IQR)	34 (20–70)	99 (49–238)	61 (29–172)
No. of obstetric complications per month: Median (IQR)	2 (0–6)	7.5 (3–20)	4 (1–15)
Number of direct obstetric complications per month: Median (IQR)	2 (0–6)	7.5 (3–20)	4 (1–15)
Stillbirth rate (per 1000 births): Median (IQR)	0 (0–5.3)	21.7 (0–35.7)	9.0 (0–28.0)
Early neonatal death rate (per 1000 live births): Median (IQR)	0 (0–0)	4.3 (0–11.5)	0 (0–8.9)
Proportion of women with obstetric complications: n/N (%)	0/190 (0%)	6/1,435 (0.4%)	6/1,625 (0.4%)
Direct obstetric complications: n/N (%)	0/180 (0%)	4/1,430 (0.3%)	4/1,610 (0.25%)
Haemorrhage: n/N (%)	0/48 (0%)	2/350 (0.6%)	2/398 (0.50%)
PPH: n/N (%)	0/21 (0%)	1/149 (0.7%)	1/170 (0.59%)
(Pre-)Eclampsia: n/N (%)	0/61 (0%)	2/390 (0.5%)	2/451 (0.44%)
Postpartum sepsis: n/N (%)	0/1 (0%)	0/63 (0%)	0/64 (0%)
Obstructed labour: n/N (%)	0/70 (0%)	0/611 (0%)	0/681 (0%)
Ruptured uterus: n/N (%)	0/0 (0%)	0/16 (0%)	0/16 (0%)
Indirect obstetric complications: n/N (%)	0/10 (0%)	2/5 (40%)	2/15 (13%)

PPH, postpartum haemorrhage.

The SBR was 25.4 per 1000 births at baseline, the median for basic and comprehensive EmOC facilities were 0 (IQR 0–5.3) and 22 (IQR 0–36), respectively. The ENND rate was 9.6 per 1000 live births; for basic and comprehensive EmOC facilities, the median was 0 (IQR 0-0) and 4.3 (IQR 0–11.5), respectively. In total (for the baseline period), there were six maternal deaths, all at Comprehensive EmOC facilities; hence, the overall iMMR was 38 per 100 000 live births. About 99% (1610/1625) of all emergency obstetric complications were direct obstetric complications, of which the majority 89% (1430) were managed at comprehensive EmOC facilities. When all direct complications at baseline were considered, the direct obstetric CFR was 0.25%. (Pre-)Eclampsia was the most common complication (n=451) at baseline, followed closely by all haemorrhage (n=398), followed by PPH (n=170) and, less commonly, postpartum sepsis (n=64).

### Effect of intervention

The 127 participating facilities were each expected to provide data for 24 months after excluding transition months, of which 1335 were preintervention and 1713 postintervention. Most HCFs provided data every month. However, there were five facilities for which data were missing for a non-transition month on 21 occasions (0.7% of total): 9 occasions in two facilities preintervention (once for a facility in district 9 and 8 times for a facility in district 10) and 12 occasions in three facilities postintervention (4 and 6 times, respectively, for two facilities in district 2, 2 times for a facility in district 7). Reasons for this included HCF shut and/or missing birth registers at time of assessment. The facility in district 10 which did not provide data eight times preintervention could not be categorised for workload and provided no data for obstetric complications. In addition, to the 37/3048 months per facility for which obstetric complication data were not obtained, data for obstetric complications were also missing on 24/3027 (0.8%) occasions (8 in the control phase). Data for deaths were additionally missing on two occasions, both in the control phase. The aggregated numbers of months per facility which provided data are indicated in table 2. Estimated components of variance are provided in online supplementary table 3.

10.1136/bmjgh-2019-001670.supp3 Supplementary data

**Table 2:** Summary statistics and estimated incidence rate ratios (IRRs) for specified primary and secondary outcomes

Outcome	Control phase		Intervention phase		Adjusted IRR‡ (95% CI)
	n* of 1335	Crude rate (ratio†)	n* of 1713	Crude rate (ratio†)	
Primary outcomes					
Stillbirths Stillbirth rate (per 1000 births)	1326	24.6 (5529/224 649)	1701	23.5 (4915/208 934)	0.97 (0.91 to 1.05)
Newborns Early neonatal death rate (per 1000 live births)	1326	9.3 (2038/219 120)	1701	9.5 (1933/204 019)	1.04 (0.92 to 1.17)
Maternal Institutional maternal mortality ratio (iMMR) (per 100 000 live births)	1311	70.9 (154/217 134)	1681	83.2 (167/200 695)	1.23 (0.80 to 1.90)
Direct obstetric CFR	1308	0.57% (124/21 767)	1677	0.62% (123/19 691)	1.15 (0.66 to 2.02)
Secondary outcomes					
iMMR for direct maternal deaths only	1311	57.1 (124/217 134)	1681	61.8 (124/200 695)	1.24 (0.79 to 1.92)
iMMR for indirect maternal deaths only	1311	13.8 (30/217 134)	1681	21.4 (43/200 695)	2.17 (0.87 to 5.40)
Obstetric case fatality rate (CFR)					
CFR—all complications (direct and indirect)	1308	0.69% (154/22 287)	1677	0.83% (166/19 902)	1.14 (0.65 to 2.01)
Indirect obstetric CFR	68	5.8% (30/520)	74	20.4% (43/211)	1.93 (1.25 to 3.01)
CFR by type of complication					
Haemorrhage§	894	0.93% (48/5147)	1111	0.81% (43/5281)	0.97 (0.60 to 1.56)
PPH§	622	2.0% (38/1860)	787	1.6% (33/2106)	0.77 (0.39 to 1.54)
Eclampsia	821	0.67% (46/6823)	913	0.86% (43/5011)	2.22 (0.96 to 5.11)
Postpartum sepsis§¶	208	4.2% (24/565)	241	5.3% (29/544)	0.95 (0.38 to 2.34)
Obstructed labour	826	0% (0/9114)	1022	0% (0/8643)	n/a
Ruptured uterus§¶	80	5.9% (6/101)	105	4.1% (7/170)	0.74 (0.21 to 2.64)
Complication rates					
Any complication	1308	10.1% (22 287/219 937)	1679	9.8% (20 090/205 398)	1.14 (1.02 to 1.27)
Haemorrhage	1305	2.4% (5148/218 881)	1660	2.6% (5338/203 774)	1.31 (1.13 to 1.52)
PPH	1307	0.85% (1863/219 414)	1678	1.04% (2133/205 918)	1.27 (0.97 to 1.65)
Eclampsia	1307	3.1% (6823/219 414)	1679	2.5% (5076/206 380)	0.95 (0.75 to 1.19)
Postpartum sepsis	1305	0.26 (568/219 232)	1679	0.27% (557/205 398)	1.86 (1.17 to 2.95)
Obstructed labour	1297	4.3% (9115/213 358)	1674	4.3% (8797/204 195)	1.12 (0.98 to 1.27)
Ruptured uterus	1309	0.05% (102/220 833)	1683	0.08% (171/206 191)	1.19 (0.66 to 2.14)

*n indicates the number of facility-month combinations for which any case was recorded, and could thus be included in the analysis.

†Unadjusted statistics aggregated across all facilities and months within phase to derive crude incidence rates.

‡Estimates are derived from mixed effects negative binomial models in which month of study, facility type and monthly numbers of births (≤30, 31–160 or >160) were included as fixed effects, with random effects for district and facility, unless indicated otherwise.

§Monthly number of births omitted from the analysis to enable estimates to be derived.

¶Facility type and random effect for facility omitted from the analysis to enable estimates to be derived.

### Stillbirth rate

**Figure 3:**
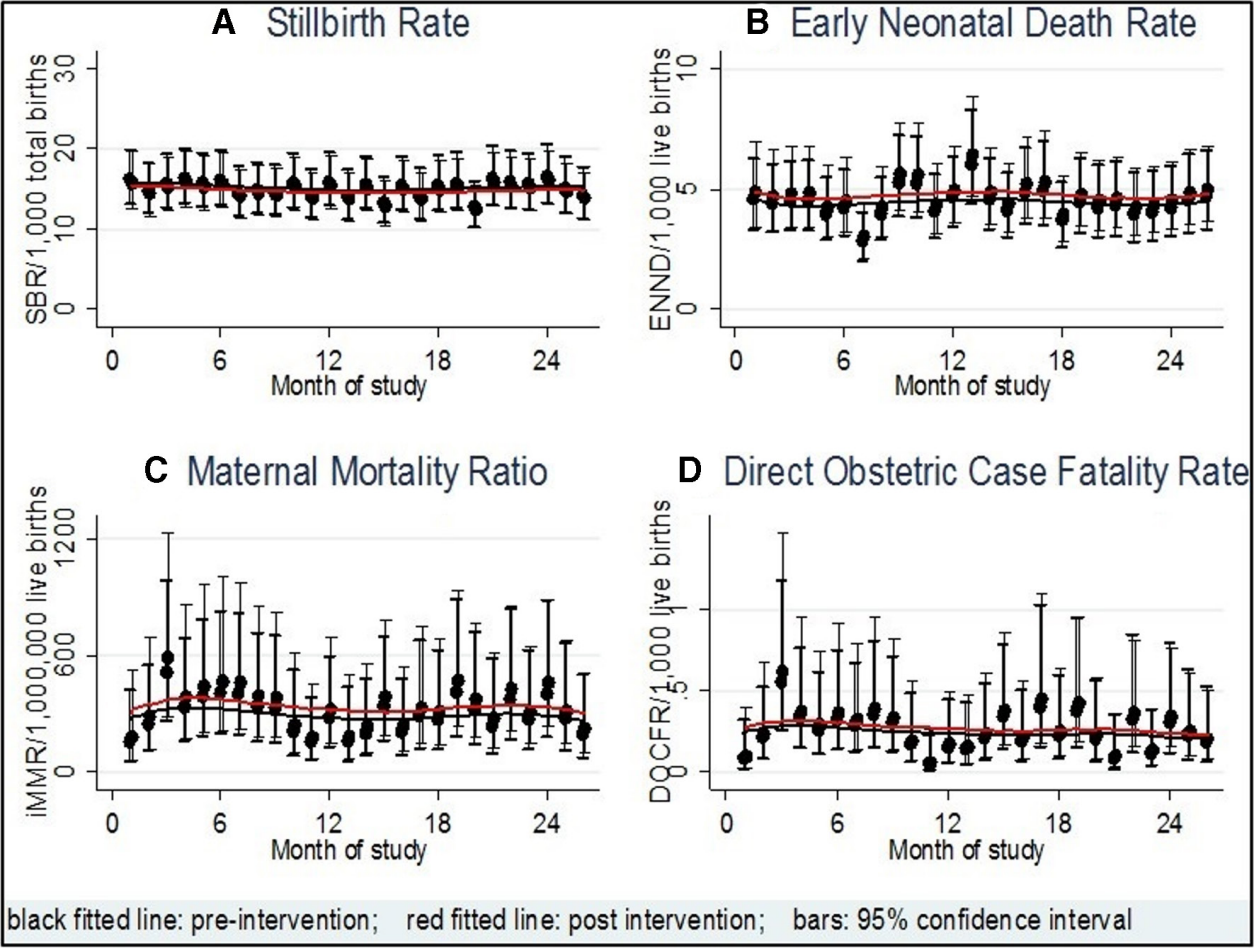
Secular trend for each primary outcome.

Over the course of the study period, there was some variation in the underlying SBR; however, there was no consistent pattern noted (figure 3A). There was a non-statistically significant 3% reduction in SBR with an adjusted (for time, facility type and workload, and clustering) IRR for the intervention of 0.97 (95% CI 0.91 to 1.05).

### Neonatal mortality

Over the course of the study period, there was no consistent pattern in the variation in the ENND rate (figure 3B). There was a non-significant increase from 9.3 to 9.5 per 1000 live births in the ENND rate, associated with the intervention, with an estimated adjusted (for time, facility type and workload, and clustering) IRR of 1.04 (95% CI 0.92 to 1.17).

### Maternal mortality and obstetric case fatality rates

Three hundred and forty-eight maternal deaths and 453 396 live births were reported (154/217 134 during the control phase and 167/200 695 during the intervention phase). No consistent pattern was seen in the underlying variation in MMR over the study period (figure 3C) except that the majority of deaths (99%) occurred at comprehensive EmOC level (see online supplementary table 4). There was no statistically significant difference in the iMMR between the intervention and control phases with an adjusted (for time, facility type and workload, and clustering) IRR of 1.23 (95% CI 0.80 to 1.90).

10.1136/bmjgh-2019-001670.supp4 Supplementary data

When split according to direct or indirect causes of maternal death, there was also no statistically significant difference for either iMMR: the adjusted (for time, facility type and workload, and clustering) IRR for direct causes was 1.24 (95% CI 0.79 to 1.92), and for indirect causes, it was 2.17 (95% CI 0.87 to 5.40).

No consistent pattern was seen in the underlying variation in direct obstetric CFR over the study period (figure 3D) with an adjusted (for time, facility type and workload, and clustering) IRR of 1.15 (95% CI 0.66 to 2.02). In contrast, the CFR for the subgroup of maternal deaths as a result of indirect complications was statistically significantly increased with an adjusted IRR of 1.93 (95% CI 1.25 to 3.01). Overall, the intervention did not have a significant impact on the obstetric CFR (adjusted (for time, facility type and workload, and clustering) IRR 1.14 (95% CI 0.65 to 2.01) and these remained below the UN standard of maximum of 1.0%.

Among the maternal deaths, 273 were due to direct obstetric causes, among the 44 871 women who had direct obstetric complications (124/21 767 during the control phase, 123/19 691 in the intervention phase and 26/3413 during the transition months). There was no statistically significant difference in the CFR for direct obstetric complications between the intervention and control phases (IRR 1.15, 95% CI 0.66 to 2.02).

At comprehensive EmOC facilities, the obstetric CFR was higher than at basic EmOC facilities but did not vary with workload.

The results were similar for CFRs for any specific complication; the CFR for ruptured uterus (5.9% control, 4.1% intervention) and postpartum sepsis were highest (4.2% control, 5.3% intervention). There was no evidence that the CFR for any complication was influenced by the intervention or the facility workload. There was evidence of higher underlying CFRs at comprehensive EmOC facilities than at casic EmOC facilities for haemorrhage and (pre)-eclampsia.

### Number of women recognised to need and receiving EmOC

The complication rate overall (number of women with complication/number of births) was 10.1% (22 287/219 937) in the control phase and 9.8% (20 090/205 398) in the intervention phase (adjusted IRR 1.14, 95% CI 1.02 to 1.27). The proportion of women recognised to require and receiving care for haemorrhage (adjusted IRR 1.31, 95% CI 1.13 to 1.52) and for postpartum sepsis (aIRR 1.86, 95% CI 1.17 to 2.95) was significantly increased.

### Subgroup effects: underlying risk factors

Facility type, facility workload and prestudy MMR were considered for inclusion in the analyses to account for potential underlying variation due to these factors. Interactions of these effects with intervention were also considered. No evidence of interaction was found for any interaction considered.

There was, however, an underlying, statistically significantly higher incidence rate for stillbirths, ENNDs, MMRs and direct obstetric CFRs at comprehensive compared with basic EmOC HCFs (see online supplementary table 4). In each case, there was no evidence that the magnitude of this difference changed with the intervention.

After accounting for facility type, no evidence was found that either the workload of facilities or the prestudy MMR affected any of the outcomes.

### Districts

To account for underlying variation between districts, district was included as a random effect in the analyses performed. Some variation was evident in the underlying SBRs in the districts participating in the study (see online supplementary figure 1), with districts 2, 9 and 10 having lower rates throughout; these differences were independent of the phase of the study. Some variation was also evident in the underlying neonatal death rates in the districts participating in the study (see online supplementary figure 2), with districts 2 and 9 (and to a lesser extent districts 7 and 10) having lower rates throughout; these differences were independent of the phase of the study.

10.1136/bmjgh-2019-001670.supp5 Supplementary data

10.1136/bmjgh-2019-001670.supp6 Supplementary data

## Discussion

### Main results

A 3% reduction in SBR was observed following training in EmOC but this was not statistically significant (IRR 0.97, 95% CI 0.91 to 1.05). In addition, there was no significant change in either the iMMR (IRR 1.23, 95% CI 0.80 to 1.90), ENND rates (ENND 1.04, 95% CI 0.92 to 1.17) or in CFR for any of the main obstetric complications (1.15, 95% CI 0.66 to 2.02). There was no evidence of a difference in effect with regard to level of HCF (comprehensive or basic) nor was workload (number of births per month) a determining factor.

Although CFR did not improve, there was a 14% increase in the proportion of women recognised to require EmOC for any complication (IRR 1.14, 95% CI 1.02 to 1.27); a 31% increase in the proportion of women recognised to require EmOC for obstetric haemorrhage (aIRR 1.31, 95% CI 1.13 to 1.52) and an 86% increase in the proportion of cases recognised to have, and managed for, postpartum sepsis (IRR 1.86, 95% CI 1.17 to 2.95).

### Interpretation of results

A systematic review by Opiyo and English^18^ concluded that there was moderate evidence that emergency neonatal training improved neonatal health outcomes in the short term. Similarly, a systematic literature review to assess effectiveness of ‘skills and drills’ training in EmOC^13^ has shown that training improves knowledge and skills,^12^ adherence to management protocols,^19–21^ improved documentation of procedures^19 22 23^ and improved health outcomes in case of the newborn with regard training in the management of shoulder dystocia.^19–23^

In a quasiexperimental study, Varghese *et al* 24 implemented EmOC skills and drills training in four intervention sites and compared knowledge/skills, diagnosis and management of EmOC with four control sites. Knowledge and skills improved with training but recognition and management of women with obstetric complications did not improve. Barriers to improved diagnosis and management identified were staff attrition and irregular supply of drugs and supplies.^24^ The most recent nine systematic reviews on the effectiveness of EmOC training programmes evaluated the effectiveness of training in one or more components of EmOC&NC. About 50% (51 out of 101) studies included in the review were interrupted time series or before–after studies and three were qualitative studies. Thirteen studies were randomised controlled trials (10 were conducted in high income and only three in low-middle and middle-income country settings). Only about a quarter of the included studies evaluated effectiveness using health outcomes and a wide range of outcome measures have been used. The review concluded that (1) there is very good evidence that healthcare providers enjoy this type of training and find it relevant to their day-to-day work and clinical settings, (2) there is also strong evidence of statistically significant improvements in knowledge and skills after training, (3) there is good evidence of improved clinical practice including adherence to protocols for care and evidence-based practice and (4) there is limited data to support that training is translated into improved health outcomes. The review recommended robust study designs. Evaluation of effectiveness at health outcome level depends on the functionality of the health system.^13^ Although there is ample evidence that training improves knowledge and skills, as well as the confidence of healthcare providers, unless the working environment is improved, this may not result in a reduction in maternal and perinatal mortality.

### Strengths and limitations of the study

Almost all previous studies of the effectiveness of evaluating EmOC training use before and after study designs and do not report health outcomes. To the best of our knowledge, this is the first study to use a stepped wedge study design and a robust randomised approach to investigate the effectiveness of EmOC training on maternal and newborn health outcomes and on stillbirths. The stepped wedge cluster randomised trial design was chosen as it is an appropriate method for assessing interventions which are introduced into ‘routine’ services and which cannot be made available to all target sites simultaneously; the design allows each study site to act as its own control, while enabling any underlying secular effect of time on the outcomes to be accounted for.^25–27^

The absence of a statistically significant improvement following EmOC for any of the primary outcomes assessed is either due to EmOC having no impact on maternal or perinatal mortality or due to the study not detecting the impact. Any further randomised evaluations would need to involve more clusters than this study and ensure that the trial design provides opportunity for all clusters to contribute data for at least one step in each state (two clusters (districts), comprising 28 HCFs only provided data for one state in the current trial) to avoid the risks associated with few clusters.^28 29^

One district acted as a pilot for the main study, with the intervention rolled out prior to the study period to test the in-principle feasibility and acceptability of the interventions and evaluation approach. The study obtained data from all but two of the facilities in the other 11 of the 12 districts planned for the study. Data capture was almost complete, with data missing for just 0.7% of occasions. Thus, the planned study size was almost achieved. No formal sample size calculation had been performed, but as part of this implementation programme, all the poorest performing districts with the highest maternal mortality were included. The duration of the study and timing of the steps was such that two of the districts did not provide data for both the preintervention and postintervention periods which likely reduced the power of the study. A longer study period would have provided more data for all facilities, as well as data for both phases in these districts, and thereby potentially reduced the standard errors and width of CIs.

All data were extracted from routine hospital records; as such, data recording errors at source were beyond the control of the project team. The outcomes used are ratios which involve counts of both the event of interest and the number at risk. Errors in the measurement/capture of either would result in inaccurate data used for analysis, but there is no reason to suspect that this would occur differentially between the two study arms.

When the study was planned, there was a general perception that this type of trial did not need to be registered.^30^ However, the trial was registered retrospectively, on the advice of KH. The registered protocol identified maternal mortality, case fatality, stillbirth and ENND rates as the outcomes of interest. During the later months of data collection and cleaning, the outcomes to be considered and the analyses to be performed were reviewed and clarified. A notable change was made (before any data were analysed) for cause-specific deaths: originally the denominator was to be those identified as having a ‘maternal near-miss’. However, the sparseness of these data rendered this approach impossible; all cases of complications were used in the denominator as a more appropriate approach to analysis of these data.

### Implications for clinical practice and research

In a quasiexperimental study, Varghese *et al* 24 implemented EmOC skills and drills training in four intervention sites and compared knowledge/skills, diagnosis and management of emergency obstetric care with four control sites. Knowledge and skills improved with training but recognition and management of women with obstetric complications did not improve. Barriers to improved diagnosis and management identified were staff attrition and irregular supply of drugs and supplies.^24^ Although there is ample evidence that training improves knowledge, skills, confidence and competence of healthcare providers, unless the working environment is improved this may not be effective in reducing maternal and perinatal deaths.^18^

When implemented as an integrated approach as part of wider health systems strengthening programmes, training of healthcare providers has been reported to be more likely to improve utilisation of maternity services and/or result in improvements in health outcomes than when such training is provided as a stand-alone programme without additional efforts to address wider health system factors.^31 32^

A multicountry survey to assess availability of the EmOC signal functions showed that despite availability of staff, in many setting the full complement of EmOC signal functions was not available. This could be due to lack of consumables, medicines and equipment.^11^ Prior to commencing the training of healthcare providers in South Africa, a baseline survey conducted in 133 HCFs in the intervention districts found that none of the 53 community health centres provided the seven signal functions of basic EmOC and only 56% (80) of the hospitals provided all nine signal functions of comprehensive obstetric care.^33^ Staff turnover (moving from the maternity ward to other areas of the hospital or moving out of healthcare) is known to be high. Losing trained staff (if not replaced) means there is generally a loss of competency and team leadership. For this study, we did not additionally assess availability or ‘stock outs’ of equipment and drugs or the degree of staff loss. This could be helpful in future studies. Finally, although there is evidence that training improves clinical practice, this was not directly assessed in this trial other than by the recorded number of women who were recognised to need and receive care.

In our experience, the evaluation of effectiveness of complex interventions for maternal health outcomes requires more robust evidence and approaches. The stepped wedge trial approach used in this study can be seen as being on the boundary of implementation research (where randomised evaluations and primary outcomes are not always standard) and a clinical trial (where randomisation and a stated primary outcome are seen as crucial). We, however, recommend a stepped wedge trial as a feasible ‘real life’ evaluation of method of greater robustness than the more commonly conducted before–after comparisons in large-scale maternal and neonatal health programmes conducted in low-income and middle-income settings.

A parallel process evaluation would have been useful but was outside the scope of this study. Programmatic indicators were used and process evaluation was conducted in other settings and reports. A national-level advisory board reviewed and discussed all aspects of implementation and sustainability. The training has since been scaled-up to other districts.

## Conclusions

In the last decade in South Africa, maternal and perinatal mortality has overall declined and is much lower than for other countries in the region. More than 95% of women give birth in a HCF and the vast majority of women with complications at the time of childbirth do access a hospital which is able to, in principle, provide comprehensive EmOC. Simulation-based ‘skills and drills’ training workshops in EmO did not result in statistically significant reductions in maternal mortality, stillbirth or ENND rates. Although CFRs did not significantly decline, with improved knowledge and skills, healthcare providers were more able to recognise women who had acute obstetric complications and commence treatment. However, to reduce mortality rates in this setting, further improvement in the quality of care is needed. It is likely that this will also require other health system factors to be addressed including availability of 24/7 highly qualified obstetric staff.
