# Supplementary material for: Effects of emergency obstetric care training on maternal and perinatal outcomes: a stepped wedge cluster randomised trial in South Africa
Source: BMJ Glob Health. 2019 Nov 10;4(6):e001670. doi: 10.1136/bmjgh-2019-001670 (PMC6861119; doi:10.1136/bmjgh-2019-001670)
Supplement: Supplementary data [file bmjgh-2019-001670supp001.pdf]

Supplementary Table 1: Outcome measures assessed for step wedge cluster randomised trial of effectiveness of training in Emergency Obstetric Care

**Outcome**	**Definition**
**Primary outcomes**	
Stillbirths<br>Stillbirth rate (SBR)	Number of still births per 1000 births
Early Neonatal Death Rate (ENNDr)	Number of facility based early neonatal deaths per 1000 live births
Institutional maternal mortality ratio (iMMR)	Number of maternal deaths (direct and indirect) per 100,000 live births
Direct Obstetric Case Fatality Rate (CFR)	Number of maternal deaths due to direct obstetric complications per 100 cases of direct obstetric complications
**Secondary outcomes**	
CFR – all complications	Number of maternal deaths due to obstetric complications per 100 cases with an obstetric complication
Indirect Obstetric CFR	Number of maternal deaths due to indirect obstetric complications, per 100 cases of indirect obstetric complications,
**CFR by type of complication**	**Number of deaths per 100 cases due to**
All Haemorrhage CFR	Abruptio, Placenta previa, PPH or Retained placenta
Postpartum Haemorrhage (PPH) CFR	PPH only (sub set of above)
(Pre-)Eclampsia CFR	(Pre-)Eclampsia
Postpartum Sepsis CFR	Sepsis in the postpartum period
Obstructed labour CFR	Obstructed labour
Ruptured uterus	Ruptured uterus
**Complication rates**	**Number of complications per 100 births due to**
Any cause	All direct and indirect complications
All Haemorrhage rate	Abruptio, Placenta previa, PPH or Retained placenta
PPH rate	PPH only (sub set of above)
(Pre-)Eclampsia rate	(Pre-)Eclampsia complications
Postpartum Sepsis rate	Postpartum sepsis
Obstructed labour rate	Obstructed labour
Ruptured uterus	Ruptured uterus
