# Supplementary material for: Effects of emergency obstetric care training on maternal and perinatal outcomes: a stepped wedge cluster randomised trial in South Africa
Source: BMJ Glob Health. 2019 Nov 10;4(6):e001670. doi: 10.1136/bmjgh-2019-001670 (PMC6861119; doi:10.1136/bmjgh-2019-001670)
Supplement: Supplementary data [file bmjgh-2019-001670supp002.pdf]

<u>Supplementary Table 2</u>: Data Quality Issues

The following corrections have been made to data in the database, further to discrepancies identified in the data

**Records affected**	**Variables changed**	**Changes made**	**Reason**
Those in which total live births was not equal with total births plus total stillbirths	Total live births	=total births – total stillbirths	Inconsistent data
All records	Total deliveries	Data not used	Data incorrectly derived from total births – usually matches even though there were some multiple births
A BEmOC facility (115) in district 2 with >160 deliveries at baseline in Feb 2014	Five obstetric complications - number of cases	Set to missing	Number of deaths reported exceeds number of cases
	Five obstetric complications - number of deaths	Set to missing	
	Total direct complications - number of cases	Values swapped	Numbers of cases was 0 but 28 deaths were reported
	Total direct complications - number of deaths		
A CEmOC facility (52) in district 3 with >160 deliveries at baseline in Aug 2014	Number of postpartum sepsis cases	Set to missing	Number of sepsis cases in all other month's totals 2; reported number of 114 is not plausible
7 records from 4 facilities	Total direct complications – number of cases	Set to missing	Missing data for the number of cases for some complications
	Total direct complications – number of deaths	Set to missing	Missing data for the number of cases for some complications
	Total complications – number of cases	Set to missing	Missing data for the number of cases for some complications
	Total complications – number of deaths	Set to missing	Missing data for the number of cases for some complications

van den Broek N, *et al. BMJ Global Health* 2019; 4:e001670. doi: 10.1136/bmjgh-2019-001670
