# Supplementary material for: Effects of emergency obstetric care training on maternal and perinatal outcomes: a stepped wedge cluster randomised trial in South Africa
Source: BMJ Glob Health. 2019 Nov 10;4(6):e001670. doi: 10.1136/bmjgh-2019-001670 (PMC6861119; doi:10.1136/bmjgh-2019-001670)
Supplement: Supplementary data [file bmjgh-2019-001670supp003.pdf]

Supplementary Table 3: Estimated components of variance for each outcome

Outcome	Log (alpha)*	Variances	
		District (95% CI)	Facility (95% CI)
**Primary outcomes**			
Stillbirths<br>Stillbirth rate (per 1,000 births)	-3.89<br>(-4.26,-3.51)	0.01<br>(0.00,0.18)	0.20<br>(0.12,0.32)
Newborns:<br>Early Neonatal Death Rate (per 1,000 live births)	-1.57<br>(-2.20,-0.94)	0.00<br>(0.00,0.00)	0.29<br>(0.17,0.49)
Maternal: Institutional maternal mortality ratio (iMMR) (per 100,000 live births)	-0.81<br>(-1.68,0.06)	0.06<br>(0.01,0.75)	0.58<br>(0.22,1.49)
Direct obstetric CFR	-0.21<br>(-0.58,0.17)	0.14<br>(0.04,0.49)	0.76<br>(0.32,1.82)
**Secondary outcomes**			
iMMR for direct maternal deaths only	-0.64<br>(-1.23,-0.05)	0.02<br>(0.00,137)	0.74<br>(0.31,1.78)
iMMR for indirect maternal deaths only	-11.2<br>(-41.5,19.0)	1.64<br>(0.47,5.69)	0.00<br>(0.00,0.00)
**Obstetric Case Fatality Rates (CFR)**			
CFR – all complications (direct & indirect)	-0.34<br>(-0.81,0.13)	0.17<br>(0.06,0.53)	0.85<br>(0.30,2.25)
Indirect obstetric CFR	-15.6<br>(-16.6,-14.7)	0.00<br>(0.00,0.00)	1.15<br>(0.15,8.62)
**CFR by type of complication**			
Haemorrhage[d]	-0.26<br>(-1.93,1.40)	0.00<br>(0.00,0.00)	0.86<br>(0.32,2.31)
PPH[d]	-0.34<br>(-1.88,1.20)	0.00<br>(0.00,0.00)	1.44<br>(0.59,3.50)
Eclampsia	-0.19<br>(-1.82,1.44)	0.00<br>(0.00,0.00)	1.07<br>(0.40,2.91)
Postpartum sepsis[d,e]	0.25<br>(-0.78,1.29)	0.52<br>(0.18,1.52)	n/a
Obstructed labour		n/a	n/a
Ruptured uterus[d,e]	0.24<br>(-1.16,1.63)	0.00<br>(0.00,0.00)	n/a
**Complication rates**			
Any complication	-1.26<br>(-1.61,-0.90)	0.10<br>(0.05,0.19)	0.45<br>(0.27,0.75)
Haemorrhage	-1.62<br>(-1.90,-1.34)	0.08<br>(0.03,0.23)	0.26<br>(0.16,0.42)
PPH	-0.88<br>(-1.06,-0.70)	0.05<br>(0.01,0.24)	0.50<br>(0.35,0.71)
Eclampsia	-0.65<br>(-0.93,-0.38)	0.10<br>(0.03,0.36)	0.85<br>(0.61,1.19)

**Outcome**	**Log (alpha)***	**Variances**	
		District (95% CI)	Facility (95% CI)
Postpartum sepsis	0.15 (-0.46,0.75)	0.20 (0.03,1.14)	1.93 (1.09,3.42)
Obstructed labour	-0.27 (-0.58,0.04)	0.08 (0.02,0.32)	1.06 (0.67,1.68)
Ruptured uterus	0.67 (0.02,1.33)	0.27 (0.06,1.23)	0.78 (0.54,1.13)

* alpha measures the magnitude of conditional over-dispersion and is a positive quantity; log(alpha) is a rescaling used in model fitting and estimation
