# Supplementary material for: Effects of emergency obstetric care training on maternal and perinatal outcomes: a stepped wedge cluster randomised trial in South Africa
Source: BMJ Glob Health. 2019 Nov 10;4(6):e001670. doi: 10.1136/bmjgh-2019-001670 (PMC6861119; doi:10.1136/bmjgh-2019-001670)
Supplement: Supplementary data [file bmjgh-2019-001670supp004.pdf]

Supplementary Table 4: Summary statistics (Crude Rate and ratio[a]), for primary outcomes, by facility type and study phase and estimated incidence rate ratios for intervention, facility type and their interaction

Outcome	Facility Type	Control phase	Intervention phase	Intervention[b]		Facility type[b]		Interaction[b]	
				Adjusted IRR (95% CI)	p-value	Adjusted IRR (95% CI)	p-value	Adjusted IRR (95% CI)	p-value
Stillbirths<br>Stillbirth rate (per 1,000 births)	Basic	7.53 (266/35,342)	8.07 (304/37,685)	0.91 (0.71,1.18)	0.50	2.60 (1.97,3.44)	<0.001	1.07 (0.85,1.35)	0.57
	Comprehensive	27.8 (5,263/189,307)	26.9 (4,611/171,249)						
Newborns<br>Early Neonatal Death Rate (per 1,000 live births)	Basic	1.56 (55/35,076)	1.50 (56/37,381)	1.02 (0.48,2.16)	0.96	5.18 (2.66,10.1)	<0.001	1.03 (0.47,2.25)	0.93
	Comprehensive	10.8 (1,983/184,044)	11.3 (1,877/166,638)						
Maternal<br>Institutional maternal mortality ratio (iMMR) (per 100,000 live births)	Basic	3.0 (1/33,422)	5.5 (2/36,328)	1.57 (0.17,14.4)	0.69	18.2 (2.8,121)	0.003	0.78 (0.09,7.01)	0.83
	Comprehensive	83.3 (153/183,712)	100.4 (165/164,367)						
Direct obstetric case fatality rate (per 100 complications)	Basic	0.03 (1/3,214)	0.07 (2/2,922)	1.11 (0.10,12.43)	0.93	13.8 (1.92,99.1)	0.009	1.04 (0.08,12.8)	0.98
	Comprehensive	0.80 (153/19,073)	0.97 (164/16,980)						

a Unadjusted statistics aggregated across all facilities and months within phase and facility type to derive crude incidence rates

b Estimates are derived from mixed effects negative binomial models in which month of study, facility type, monthly numbers of births (≤30, 31-160 or >160) and the interaction between intervention and facility type were included as fixed effects, with random effects for District and facility
