# Supplementary figures and images for: Effects of emergency obstetric care training on maternal and perinatal outcomes: a stepped wedge cluster randomised trial in South Africa
Source: BMJ Glob Health. 2019 Nov 10;4(6):e001670. doi: 10.1136/bmjgh-2019-001670 (PMC6861119; doi:10.1136/bmjgh-2019-001670)

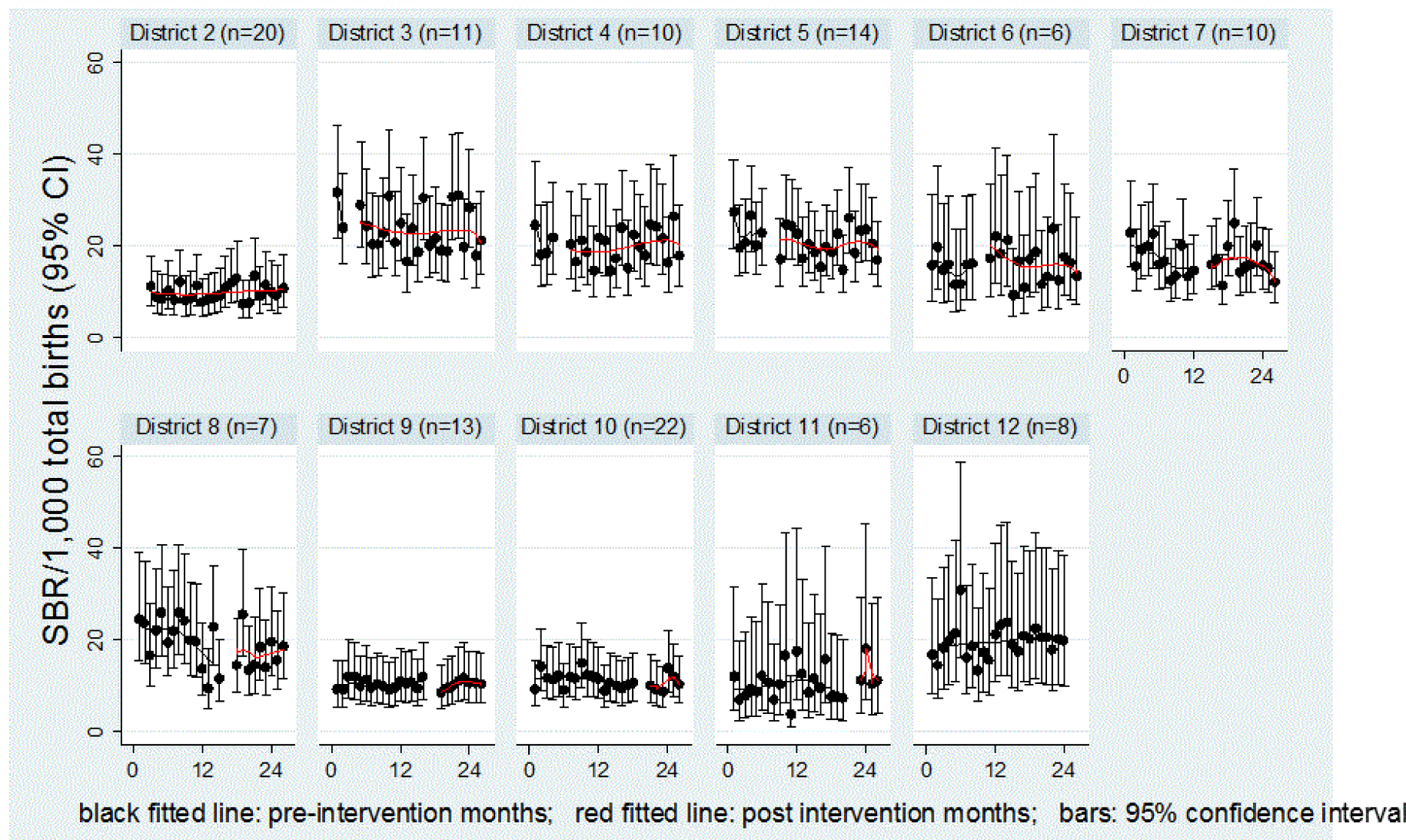

Supplement: Supplementary data [file bmjgh-2019-001670supp005.pdf]

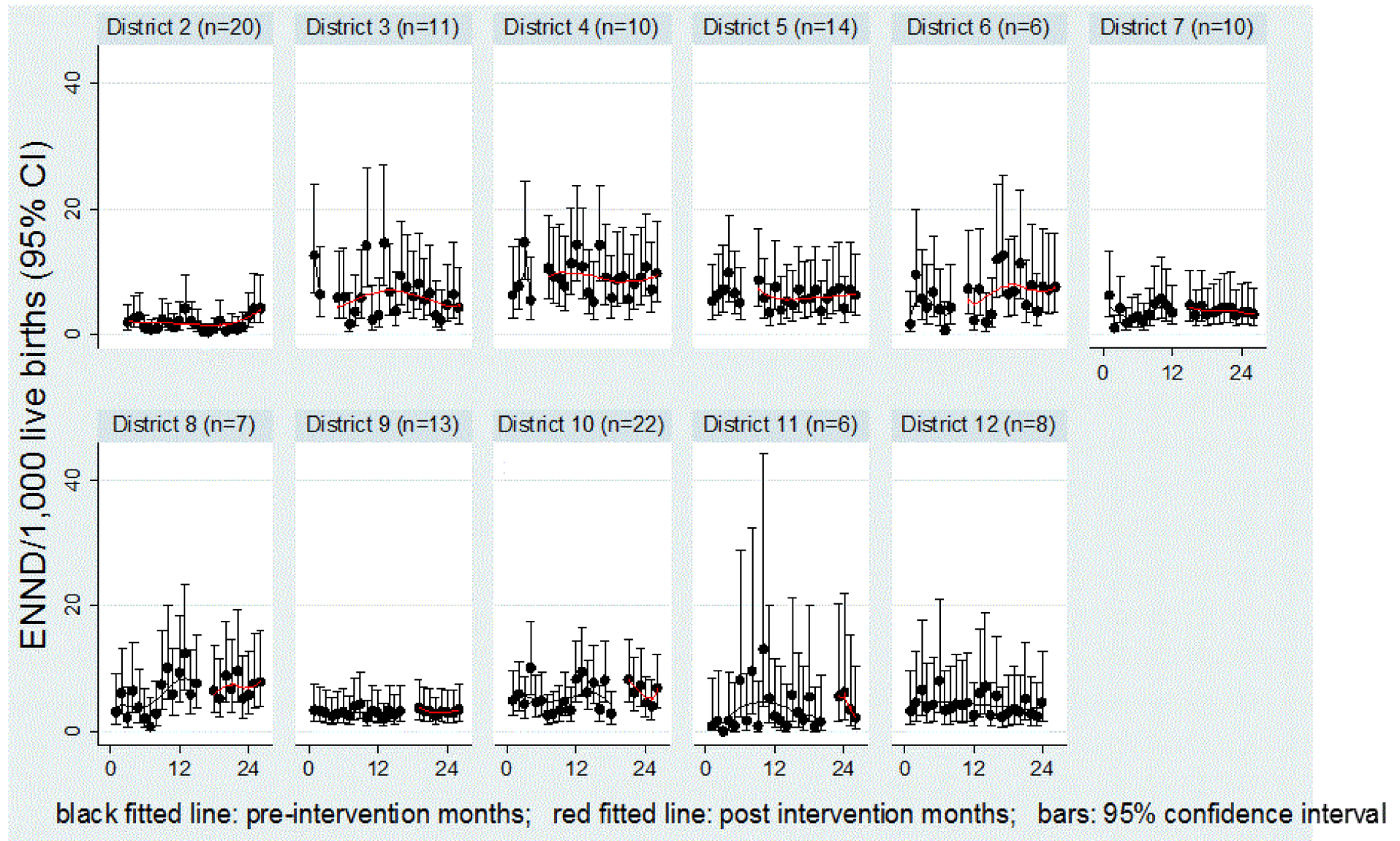

Supplement: Supplementary data [file bmjgh-2019-001670supp006.pdf]
